# Individual differences in eyewitness accuracy across multiple lineups of faces

**DOI:** 10.1186/s41235-018-0121-8

**Published:** 2018-08-08

**Authors:** Andrew J. Russ, Melanie Sauerland, Charlotte E. Lee, Markus Bindemann

**Affiliations:** 1 School of Psychology, University of Kent, Canterbury, CT2 7NP UK; 2 Department of Clinical Psychological Science, Section Forensic Psychology, Maastricht University, Maastricht, The Netherlands

**Keywords:** Eyewitness identification, Face recognition, Multiple lineups, Individual differences

## Abstract

Theories of face recognition in cognitive psychology stipulate that the hallmark of accurate identification is the ability to recognize a person consistently, across different encounters. In this study, we apply this reasoning to eyewitness identification by assessing the recognition of the same target person repeatedly, over six successive lineups. Such repeat identifications are challenging and can be performed only by a proportion of individuals, both when a target exhibits limited and more substantial variability in appearance across lineups (Experiments 1 and 2). The ability to do so correlates with individual differences in identification accuracy on two established tests of unfamiliar face recognition (Experiment 3). This indicates that most observers have limited facial representations of target persons in eyewitness scenarios, which do not allow for robust identification in most individuals, partly due to limitations in their ability to recognize unfamiliar faces. In turn, these findings suggest that consistency of responses across multiple lineups of faces could be applied to assess which individuals are accurate eyewitnesses.

**Electronic supplementary material:**

The online version of this article (10.1186/s41235-018-0121-8) contains supplementary material, which is available to authorized users.

## Significance

Eyewitness identifications are crucial for police investigations to determine the perpetrators of crime. Many eyewitnesses make perfectly accurate identifications, but many are also prone to error. In criminal investigations, the difficulty therefore arises in differentiating accurate from inaccurate eyewitnesses. Whereas eyewitnesses typically attempt to identify a perpetrator once, theories of face recognition in cognitive psychology stipulate that accurate identification is characterized by the ability to recognize the same face *repeatedly*. However, such theories are based on the recognition of familiar people, who are well-known to an observer, rather than the recognition of unfamiliar people, which typically gives rise to error in eyewitness scenarios. We therefore tested the ability of observers to identify an unfamiliar person repeatedly in an eyewitness paradigm to determine the extent to which this is possible. We argue that an observer’s ability to identify a target person repeatedly should increase our confidence that an accurate eyewitness identification has been made by this individual. Across three experiments, we find that only a minority of observers can act on a target consistently. These findings suggest that we should measure the *degree* of familiarity that an eyewitness has gained with a target to better assess their identification accuracy and indicate that this could be achieved at an individual level with multiple lineups of faces.

## Background

In criminal investigations, eyewitnesses are routinely required to identify a previously seen perpetrator from a police lineup. In the UK alone, tens of thousands of identity lineups are administered for this purpose every year (e.g. http://www.viper.police.uk). Under these circumstances, many individuals make perfectly accurate eyewitness identifications. However, eyewitness misidentifications are also frequently made, whereby innocent people in a lineup are mistaken for a perpetrator (e.g. Memon, Havard, Clifford, Gabbert, & Watt, 2011; Slater, 1994; Wright & McDaid, 1996). For investigators, the difficulty therefore arises in differentiating individuals that are accurate from those that are inaccurate eyewitnesses.

One approach under investigation in psychology to address this problem is a multiple-lineup method. In this method, eyewitnesses are required to identify a perpetrator repeatedly, but from different person aspects that might have been observed at a crime scene, such as the face, body, voice, clothing, or accessories (Lindsay, Wallbridge, & Drennan, 1987; Pryke, Lindsay, Dysart, & Dupuis, 2004; Sauerland & Sporer, 2008; Sauerland, Stockmar, Sporer, & Broers, 2013). These studies show that the selection of the same identity from different lineup combinations can be used to assess the likelihood that a correct target selection has been made. Sauerland and Sporer (2008) found, for example, that identification of a suspect’s body from a lineup indicated only a .60 probability that the identified person was, in fact, guilty. However, this number rose to .91 when the separate identification of body and face cues, from two different lineups, were considered together.

These findings illustrate the promise that multiple lineups hold for assessing the accuracy of individual eyewitnesses. However, the results of these studies are curtailed by the poor identification accuracy for some person aspects. For example, in studies that have examined multiple-lineup procedures, correct identifications of voices were obtained on only 27% of trials (Pryke et al., 2004) and this number was lower still for bodies and accessories, at 18% and 11%, respectively (Sauerland & Sporer, 2008). In comparison, identification accuracy was much higher for frontal views of faces, at 72% (Pryke et al., 2004) and 61% (Sauerland & Sporer, 2008), and combinations of other person aspects with such frontal face portraits were most useful for diagnosing eyewitness accuracy.

In this study, we sought to investigate this face advantage further, by exploring a new variant of this procedure in which multiple lineups *only* comprised faces. This manipulation makes good sense given the comparatively high recognition accuracy for faces in previous multiple-lineup studies, but it also has a strong theoretical grounding in the face perception literature. According to cognitive theories of face processing (e.g. Bruce & Young, 1986; Burton, Bruce, & Johnston, 1990; Burton, Jenkins, Hancock, & White, 2005; Haxby, Hoffman, & Gobbini, 2000; Schweinberger & Burton, 2003), the successful recognition of familiar people, such as family, friends, or colleagues, is highly robust and can be triggered by *any* instance of their face. The ultimate hallmark of accurate person identification is therefore the ability to recognize the same person’s face *repeatedly*, across many different encounters.

In line with this research, eyewitness identification errors are made rarely when the perpetrator is someone that is already known to a witness (e.g. Memon et al., 2011). Familiarity with a face is, however, a continuum (e.g. Clutterbuck & Johnston, 2002, 2004), which reflects the exposure duration to an identity (e.g. Bornstein, Deffenbacher, Penrod, & McGorty, 2012; Memon, Hope, & Bull, 2003; Roark, O’Toole, Abdi, & Barrett, 2006) as well as the variability in a person’s appearance across different exposures (Andrews, Jenkins, Cursiter, & Burton, 2015; Burton, Kramer, Ritchie, & Jenkins, 2016; Dowsett, Sandford, & Burton, 2016; Murphy, Ipser, Gaigg, & Cook, 2015). Eyewitnesses who are initially *unfamiliar* with a perpetrator cannot acquire strong familiarity (e.g. as they would in the case of the faces of family, friends, celebrities) with this person’s face at a crime scene. As a consequence, identification of such unfamiliar people can be rather difficult, even under best-possible conditions (e.g. Bruce, Henderson, Greenwood, Hancock, Burton, et al., 1999; Henderson, Bruce, & Burton, 2001; Megreya & Burton, 2006, 2008).

Another factor appears to determine eyewitness performance, as identification accuracy varies even when exposure to a perpetrator is held constant across participants. One possibility is that this reflects individual differences in the ability to recognize unfamiliar faces, whereby some individuals are naturally equipped better than others for this task (e.g. Wilmer, Germine, Chabris, Chatterjee, Williams, et al., 2010; Zhu, Song, Hu, Li, Tian, et al., 2010). Support for this reasoning comes from studies that have revealed correlations of eyewitness accuracy with tests of face recognition (e.g. Geiselman, Tubridy, Blumkin, Schroppel, Turner, et al., 2001; Hosch, 1994; Morgan, Hazlett, Baranoski, Doran, Southwick, et al., 2007). Bindemann, Brown, Koyas, and Russ (2012) showed, for example, that eyewitness identification accuracy for the perpetrator of a staged crime correlated with performance on a laboratory test of face recognition, in which observers had to select newly learned face targets from identity lineups. So far, however, these studies have only examined this link for a single eyewitness identification.

This is an important issue as there is evidence to suggest that, in contrast to familiar face recognition, the *repeated* recognition of unfamiliar faces might also be particularly difficult. Studies that speak to this issue have focused primarily on unfamiliar face *matching* tasks, in which observers have to decide whether side-by-side photographs of two unfamiliar faces depict the same person or different people. For example, in these studies observers often decide that a face pair depicts two different people in one block of trials, but classify this pair as depicting the same person in a subsequent block (Alenezi & Bindemann, 2013; Alenezi, Bindemann, Fysh, & Johnston, 2015) or on a different day (Bindemann, Avetisyan, & Rakow, 2012). When these effects are assessed at a group level, by averaging performance across participants, identification accuracy declines with each repetition of the face pairs. However, observers also exhibit broad inter-individual (e.g. Burton, White, & McNeill, 2010) and intra-individual differences (Bindemann et al., 2012).

The differences between familiar and unfamiliar face processing, *and* the individual differences that are observed in unfamiliar face matching tasks, have important implications for our understanding of eyewitness identification, for two reasons. First, a lineup task is essentially a *test of the familiarity* that an eyewitness has gained with a perpetrator’s appearance at a crime scene. Following only a brief exposure to a perpetrator (as is often the case at crime scenes), one should therefore expect that eyewitnesses generally have relatively poor memory for a perpetrator’s facial appearance and identification of this person should be taxing. Second, this issue should be compounded by inter- and intra-individual differences in the ability to process faces. Thus, observers at the lower end of face processing ability should be more prone to make errors in a single eyewitness identification and those who tend to be more inconsistent in their identification decisions should also be less capable of identifying a perpetrator repeatedly. In turn, an eyewitness’ ability to correctly recognize an unfamiliar perpetrator across multiple instances should greatly increase one’s confidence in the accuracy of their identification.

Exploring this issue will reveal new information about the robustness of facial representations that eyewitnesses hold of a perpetrator. We therefore assessed the extent to which individual eyewitnesses can identify the same person’s face repeatedly from different identity lineups and, equally, whether they can consistently indicate the absence of a target when he or she is not included in a lineup. To fully explore this question, it is important to compare consistent target identifications with the repeated selection of other, non-target lineup identities. Outside of the laboratory, an identity lineup always includes a suspect, but this person may be the sought-after perpetrator of a crime or an innocent person. The purpose of a lineup is essentially to determine whether a witness selects the suspect, thereby seemingly confirming them as the target, or does not select this individual. The remaining faces act as “fillers” that are known innocents that would not be charged if they were selected by an eyewitness. To determine the extent to which observers might repeatedly identify the same non-target face in a multiple-lineup procedure, one could therefore replace the target with another identity that acts as a designated innocent suspect. By comparing repeated target identifications with selections of the innocent suspect, it would then be possible to determine whether the consistency of the responses of individual observers across multiple lineups can dissociate correct from incorrect eyewitness identifications.

While this approach has obvious applied value, the designation of innocent suspects poses problems in experimentation (Pryke et al., 2004; Sauerland & Sporer, 2008). In police investigations, suspects are arrested on the basis of their similarity to a witness’ description. However, it can be difficult to establish the perceived similarity of targets and suspects in advance. Different strategies for designating innocent suspects and lineup fillers appear to influence eyewitnesses’ identification decisions (Lindsay, Martin, & Webber, 1994; Luus & Wells, 1991; Wells, Rydell, & Seelau, 1993), but the study of such strategies has also yielded inconsistent results (e.g. Darling, Valentine, & Memon, 2008; Tunnicliffe & Clark, 2000). In addition, studies of unfamiliar face matching demonstrate that people vary considerably in how they detect the resemblance of faces in person identification tasks (e.g. Bindemann et al., 2012; Burton et al., 2010).

In light of these problems, we adopted a different approach. Instead of pre-selecting a designated suspect, this identity was defined *a posteriori*. We provide two contrasting approaches for this analysis. For the first approach, we assess identifications of the non-target identity that was selected *first* by an eyewitness in the multiple-lineup procedure. This approach minimizes data loss by including all incorrect eyewitnesses in the analysis and provides a “worst case” scenario by comparing consistent target selections with the greatest possible number of the corresponding non-target identifications. With a second approach, we focus on the non-target that was selected most often as the target by all observers during the course of the entire experiment. This “worst non-target” approach provides the highest number of comparison identifications for the target when these are defined by only a single non-target identity (for similar approaches, see, e.g. Pryke et al., 2004; Sauerland & Sporer, 2008). The consistency of these non-target selections across lineups served to contextualize the extent of consistent target selections.

## Experiment 1

To investigate the extent to which individuals can repeatedly identify a target in a multiple-lineup procedure with faces, we conducted a field experiment in which pedestrians in a city center were approached by a target person under the pretense of requiring route directions to a local landmark. Shortly after this exchange, pedestrians were approached by an experimenter and asked to attempt to identify the just-seen target. For this purpose, six successive identity lineups of faces were shown, comprising a mixture of three target-present and three target-absent lineups. Our aim here was to assess the extent to which individual observers could identify the target person repeatedly.

## Method

### Participants

Forty pedestrians in a town center (23 women, 17 men), consisting of students and young professionals with a mean age of 22 years (range = 14–36 years, *SD* = 4.7), took part in this experiment. These participants agreed to take part in the experiment once they had been made aware of the true purpose of the initial interaction with the target and had provided informed consent to continue further. Approximately 75% of people originally approached agreed to participate. All participants reported normal or corrected-to-normal vision.

### Materials

The faces of 12 people were used for lineup construction. These consisted of the target and 11 non-target identities. All of the non-targets fitted the general description of the referring target (Wells et al., 1993), as determined in two pilot studies with 20 mock witnesses. For each identity, three color face photographs were collected with the same camera equipment, which showed these persons in a frontal view with a neutral expression. These photographs were standardized by cropping clothing and background. Similar to face-matching studies, these images were taken on the same day to eliminate transient differences in age, facial hair, and so forth (e.g. Bruce et al., 1999; Burton et al., 2010). All of the resulting face images measured approximately 5 (W) × 7.5 (H) cm.

**Fig. 1:**
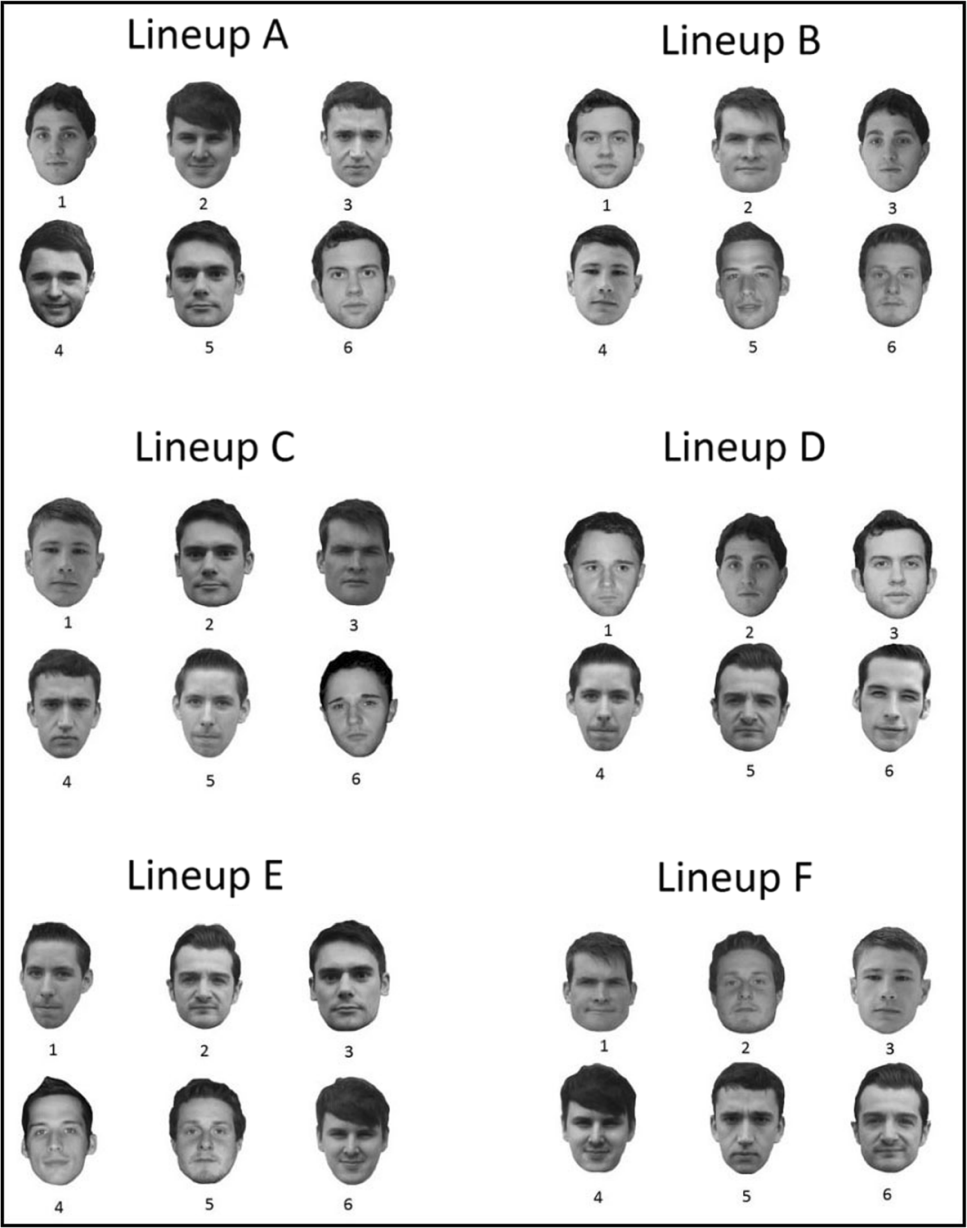
Illustration of the face images in Experiment 1, depicting target-present lineups (**a**, **c**, **e**) and target-absent lineups (**b**, **d**, **f**)

These images were then used to construct three target-absent and three target-present lineups. Effective lineup sizes were calculated using Tredoux’s *E*s and were determined to be in the range of 3.6–5.1 identities (Tredoux, 1998, 1999). Each lineup therefore consisted of six faces, which were arranged in two rows of three pictures. The target and non-target faces were distributed across these lineups, so that none of the identities appeared more than once in any of the lineups and not more than once in any of the locations across these lineups. Furthermore, no two lineups contained any more than three of the same identities. However, each of the 11 non-target identities appeared alongside the target at least once. In this way, all identities were presented three times over the course of the six lineups. Thus, it was not possible for participants to determine the identity of the target across the six lineups by virtue of this person appearing with greater frequency than the non-target faces. The lineups can be seen in Fig. 1.

### Procedure

The target, a 32-year-old Caucasian man, approached pedestrians in the center of a Dutch town to ask for directions. In these interactions, the target wore the same clothing throughout the testing period and kept the conversation as similar across participants as possible. These interactions lasted approximately 1 min. Typically, the approached pedestrian would look at the target several times during this time period. If the interaction did not follow this pattern, pedestrians were not approached again for the subsequent identification task. This was the case for approximately 25% of the approached pedestrians.

After an interval of approximately 1 min, pedestrians were approached by an experimenter, who was positioned up-street of the initial interaction with the target. At this stage, the purpose of the experiment was explained and informed verbal consent for further participation was obtained. Participants were then presented with six successive lineups, which were shown in a random order. They were told there was an equal chance that the target would be present or absent in a lineup. They were asked to attempt to identify the target when he was present or to declare his absence when he was not. Once a lineup had been completed, it was moved out of view of the participant before the next lineup was presented. No time limit was given for the identification task.

## Results

### Accuracy for individual lineups

**Fig. 2:**
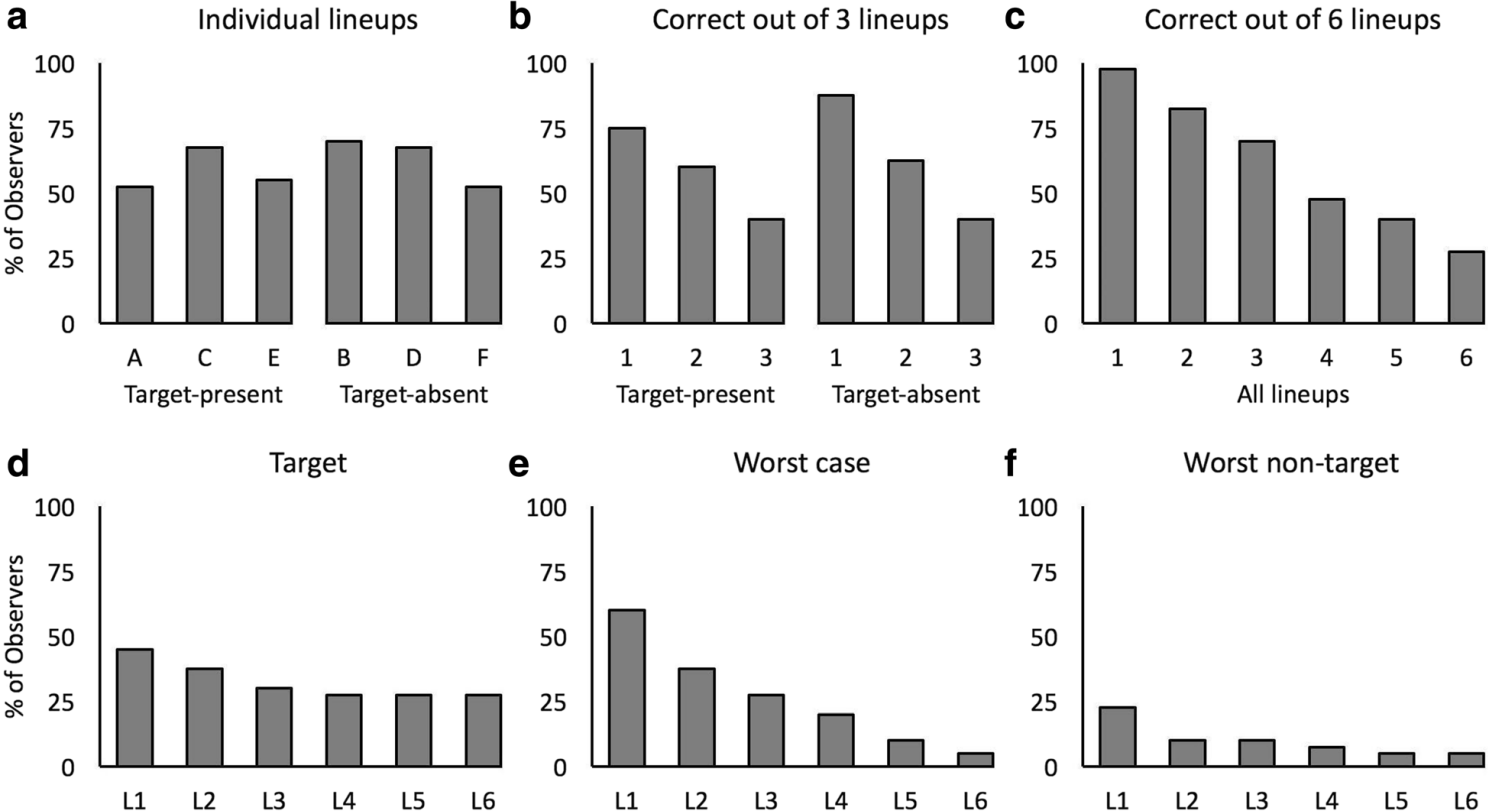
Illustration of observers’ responses in Experiment 1, showing hits for individual target-present and correct rejections for individual target-absent lineups (**a**), the number of hits and correct rejections out of three lineups (**b**), and the combined number of hits and correct rejections out of six lineups (**c**). In addition, observers’ combined hits and correct rejections were analyzed by adhering to the order in which the six lineups were seen, for the target (**d**), the worst case analysis (**e**), and for the worst non-target (**f**)

Identification accuracy was calculated separately for each of the six lineups. To simplify the results, all analyses were based on hits (i.e. the correct identification of a target from a target-present lineup) and correct rejections (the correct response that a target-absent lineup did not contain the target; note that the full dataset is provided as Supplementary Material). Hits and correct rejections are presented in Fig. 2a for each individual lineup. Note that these data refer to the specific lineups depicted in Fig. 1. Thus, the order in which these lineups were encountered by individual observers is not preserved in Fig. 2a.

These data show that observers identified the target (hits) in 53–68% of trials. Similarly, correct rejections for target-absent lineups were in the range of 53–70%. A series of Wilcoxon signed-rank tests (with alpha corrected to .05/3 = .017 for three comparisons) showed no reliable differences in hits between the three target-present lineups or between correct rejections for the three target-absent lineups. A summary of these statistical comparisons is provided in Table 1.

**Table 1:** A summary of statistical comparisons (Wilcoxon signed-rank test) for individual lineup accuracy and for accuracy out of three lineups in Experiment 1

Target-present			Target-absent		
Lineup	Accuracy	Wilcoxon	Lineup	Accuracy	Wilcoxon
Individual lineups					
A vs C	53% vs 68%	Z = 1.90, *p* = .058	B vs D	70% vs 68%	Z = 0.33, *p* = .739
C vs E	68% vs 55%	Z = 1.90, *p* = .059	D vs F	68% vs 53%	Z = 1.50, *p* = .134
A vs E	53% vs 55%	Z = 0.30, *p* = .763	B vs F	70% vs 53%	Z = 1.94, *p* = .052
Correct out of 3 lineups					
1 vs 2	75% vs 60%	Z = 2.45, *p* = .014^a^	1 vs 2	88% vs 63%	Z = 3.16, *p* = .002^a^
2 vs 3	60% vs 40%	Z = 2.83, *p* = .005^a^	2 vs 3	63% vs 40%	Z = 3.00, *p* = .003^a^
1 vs 3	75% vs 40%	Z = 3.74, *p* < .001^a^	1 vs 3	88% vs 40%	Z = 4.36, *p* < .001^a^

^a^Significant at *p* < .017, with alpha at .05 corrected for 3 comparisons

### Consistent lineup decisions in any order

We then analyzed these data in several ways to investigate the extent to which individuals repeatedly acted on the target in these lineups. First, we calculated the percentage of observers that identified the target from only one, two, or all three target-present lineups. Similarly, we explored how many observers repeatedly noticed the absence of the target correctly, by calculating the percentage of observers that recorded correct lineup rejections for only one, two, or three target-absent lineups. These data are provided in Fig. 2b. For hits, a series of Wilcoxon signed-rank tests (with alpha corrected to .05/3 = .017 for three comparisons) showed that most observers could identify the target in one lineup, but fewer identified the target twice or three times (for a summary of the comparisons, see Table 1). Similarly, most observers correctly rejected one target-absent lineup, but fewer observers made such decisions for two, or all three target-absent lineups.

Finally, we also conducted this analysis by collapsing across all lineups, as shown in Fig. 2c. This analysis demonstrated that 98% of observers acted correctly on the target at least once, but only 28% of observers did so on all six trials, *Z*(1, *N* = 40) = 5.29, *p* < .01. Taken together, these results demonstrate that the majority of observers can appear accurate on a single lineup, but only about one in four individuals was consistently correct across all lineups.

### Consistent lineup decisions in actual order

To further examine the extent to which individual observers can repeatedly act correctly on a target, we also assessed the consistency of their responses as a function of the order in which the lineups were shown. For this purpose, the data were recoded into correct and incorrect responses irrespective of target-presence and a consistent-accuracy score was determined for each lineup. This captures the extent to which observers made a correct response on the *first* of the lineups and then carried on to do so without interruption on subsequent successive trials. To illustrate, consider an observer that made a correct identification on all trials except the third. In this case, consistent accuracy was calculated by scoring only the first two trials as correct and none of the subsequent trials. Figure 2d shows the cross-subject means of the percentage accuracy of these responses. These data reveal that accuracy was at 45% in Lineup 1. However, the proportion of consistently accurate responses declined gradually with each additional identity lineup, to only 28% in Lineup 6. Wilcoxon signed-rank test demonstrated that this drop in performance was reliable, *Z*(1, *N* = 40) = 2.65, *p* < .01.

### “Worst case” analysis

To contextualize repeated lineup decisions to the target, we analyzed the repeated selection of non-target lineup identities. To create this contrast, observers’ responses were recoded if they had selected a non-target in any of the lineups. In these cases, the first non-target that was selected by an observer across the six lineups was adopted as the “target” identity for that individual. All responses to lineups that preceded or followed this non-target selection were then recoded accordingly. For example, if observers previously or subsequently rejected a lineup in which this non-target was not present, then this was treated as a correct rejection (regardless of the presence of the actual target identity). Or if a non-target selection in, say, Lineup 1 was followed by a selection of the actual target in Lineup 2, then the incorrect response to the first lineup was recoded as a “correct identification” and the correct response to the second lineup was recoded as an “identification error” for the purposes of this analysis. Recoding the data in this way allowed for a comparison between consistent target identifications and consistent identifications of non-target identities.

These data are provided in Fig. 2e and show that non-target identifications were initially high, at 60%, in the first lineup. However, consistent decisions to the same non-target identity were made by only 5% of observers across all six lineups. A Wilcoxon signed-rank test comparing non-target selections for the first lineup with consistent non-target selections by the sixth lineup showed a significant decrease over the course of the experiment, *Z*(1, *N* = 40) = 4.69, *p* < .01. In addition, target (45%) and non-target selections (60%) were not reliably different for the first lineup seen, *Z*(1, *N* = 40) = 0.97, *p* = .33, but consistent target selections (28%) were more frequent than non-target selections (5%) by the sixth lineup, *Z*(1, *N* = 40) = 2.50, *p* < .05.

### “Worst non-target” analysis

In addition to the “worst case” analysis, which was based on *any* of the non-target identities that were initially mistaken as the target, we also conducted a “worst non-target” analysis. For this purpose, we first identified the most frequently chosen non-target identity across all the participants. We then recalculated the lineup scores of each individual participant by adopting this identity as the target. These data are provided in Fig. 2f and show that these non-target selections were made by only 23% of observers in the first lineup and fell to 5% by the sixth lineup, *Z*(1, *N* = 40) = 2.65, *p* < .01. These worst non-target selections were less numerous than target selections after one lineup (45% vs 23%), *Z*(1, *N* = 40) = 2.07, *p* < .05, and after all six lineups (28% vs 5%), *Z*(1, *N* = 40) = 2.50, *p* < .05.

## Discussion

In this experiment, eyewitness identifications were error-prone for single lineups. For example, correct identifications of the target were made by 53–68% of observers for individual lineups. These identification rates are similar to previous research that has employed six-person lineups (e.g. 61% in Sauerland & Sporer, 2008; 47% in Steblay, Dysart, Fulero, & Lindsay, 2003) and demonstrate the difficulty of this task. However, a single lineup does not provide information about the *consistency* with which individual eyewitnesses can act on the same target. Experiment 1 shows that, when eyewitness accuracy is assessed across six lineups of faces, many individuals cannot make correct decisions for the same target consistently. In the current experiment, this behavior was such that in comparison to the 98% of observers who made a correct decision to at least *one* of the lineups, and the 45% of observers who made a correct decision to the *first* lineup, only 28% of observers acted correctly on *all* six lineups. The same pattern was evident when responses were broken down by the three target-present and the three target-absent lineups. Taken together, this demonstrates that repeated eyewitness identifications of an unfamiliar target are difficult. Applying theorizing from familiar face recognition (e.g. Bruce & Young, 1986; Burton et al., 2005), this indicates that many eyewitnesses have limited cognitive representations of perpetrators’ faces.

In turn, however, these data also indicate that of the 45% of participants who initially made a correct lineup decision, many also acted consistently on this target across all six lineups, comprising 28% of all observers. These data are contextualized by an analysis of incorrect identification decisions. For example, we found that only 5% of observers based their identification decisions consistently on the same non-target across all six lineups. This indicates that identification decisions across multiple lineups of faces could provide a potential index of eyewitness accuracy, whereby individuals with consistent identification responses are much more likely to have acted on a target than a non-target.

## Experiment 2

In Experiment 1, consistent target decisions across all six lineups were made by 28% of all observers, but 5% of observers also made similarly consistent decisions to a non-target. However, these results were obtained with same-day, same-camera photographs for each identity. Thus, it is possible that observers were able to repeatedly identify the target and non-targets in part due to similarity in their appearance across the lineups. To explore this issue, Experiment 2 utilized three very different types of photographs for each facial identity, comprising a standardized image, a picture from a photo-identity card, and a completely uncontrolled photograph from the profile of a social networking site. The question of main interest here concerned the extent to which consistent identifications of the target, and of non-targets, remained possible under the additional variation that was introduced by these distinct image categories.

## Method

### Participants

As in Experiment 1, pedestrians were approached by the target male in the center of a Dutch town to ask for route directions. If these pedestrians did not look at the target several times during this interaction, then they were not approached again for the subsequent identification task. This was the case for approximately 25% of the approached pedestrians. Forty pedestrians (23 women, 17 men), consisting of students and young professionals with a mean age of 20 years (range = 13–35 years, *SD* = 4.5), clearly looked at the target during the initial interaction and therefore took part in the subsequent identification task. As in Experiment 1, these participants agreed to take part once they had been made aware of the true purpose of the initial interaction with the target and had provided informed consent to continue further. All participants reported normal or corrected-to-normal vision. None had participated in the preceding experiment.

### Materials and procedure

**Fig. 3:**
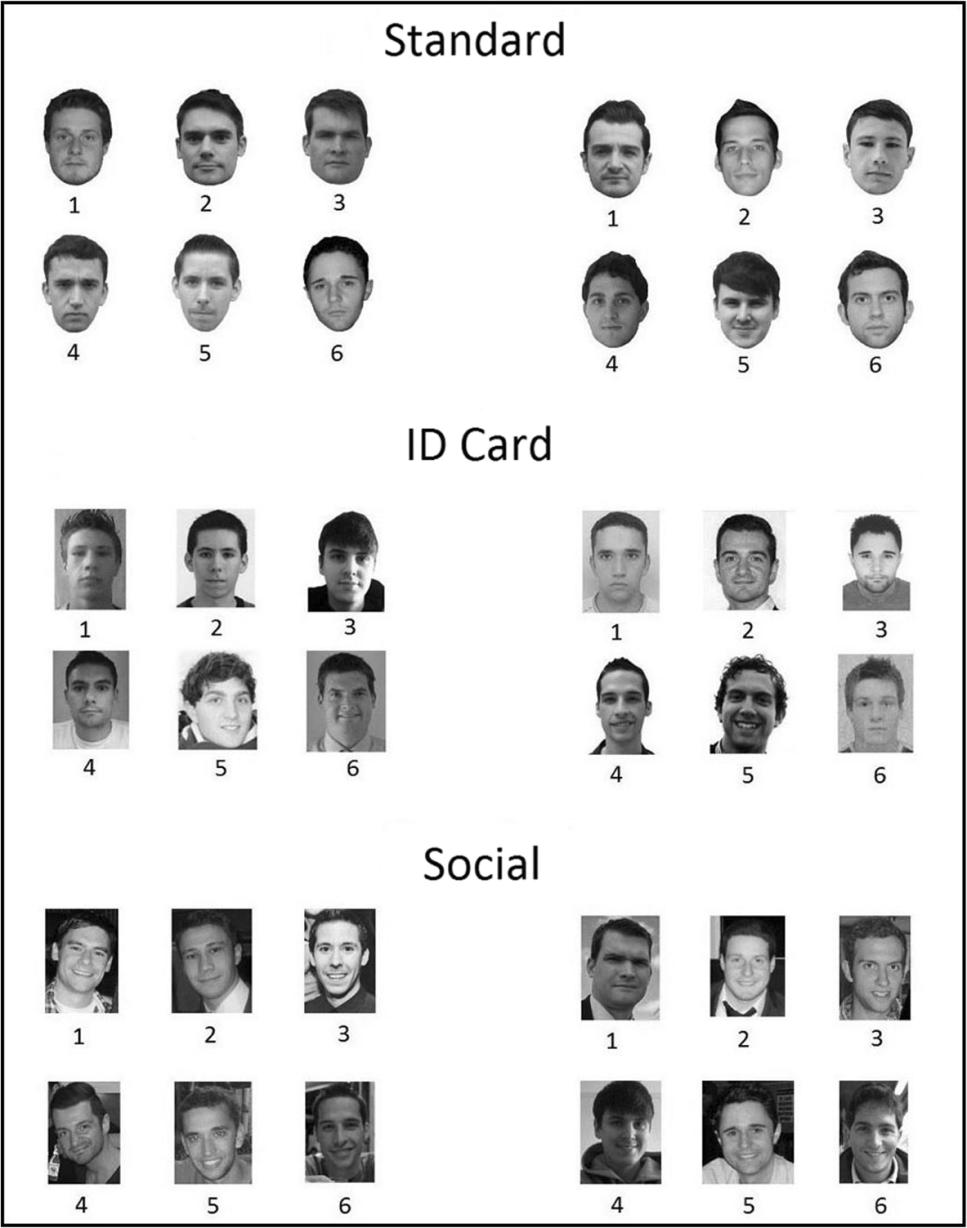
Illustration of the face images in Experiment 2, depicting target-present lineups (*left*) and target-absent lineups (*right*)

The same target and non-target identities as in Experiment 1 were used to construct the lineups. Three photographs were used for each of these identities, which comprised a standardized photograph (from Experiment 1), a photograph from a student identity-card, and a profile picture from a popular social networking site. The standardized and social face images measured approximately 5 (W) × 7.5 (H) cm but the dimensions of the identity-card images were smaller, at 2.5 (W) × 3.5 (H) cm. Due to the constraint imposed by having three image categories, only five of the 11 non-target identities could occur alongside the target in the target-present lineups of each image category. Non-targets were selected randomly to appear alongside the target in these lineups, with the constraint that none of the non-target identities occurred in the same lineup position more than once and no two lineups contained more than three of the same identities. As before, however, each lineup member contributed three images, so all identities appeared to the participant three times over the course of the six lineups. These images are illustrated in Fig. 3. The procedure was identical to Experiment 1.

## Results

### Accuracy for individual lineups

**Fig. 4:**
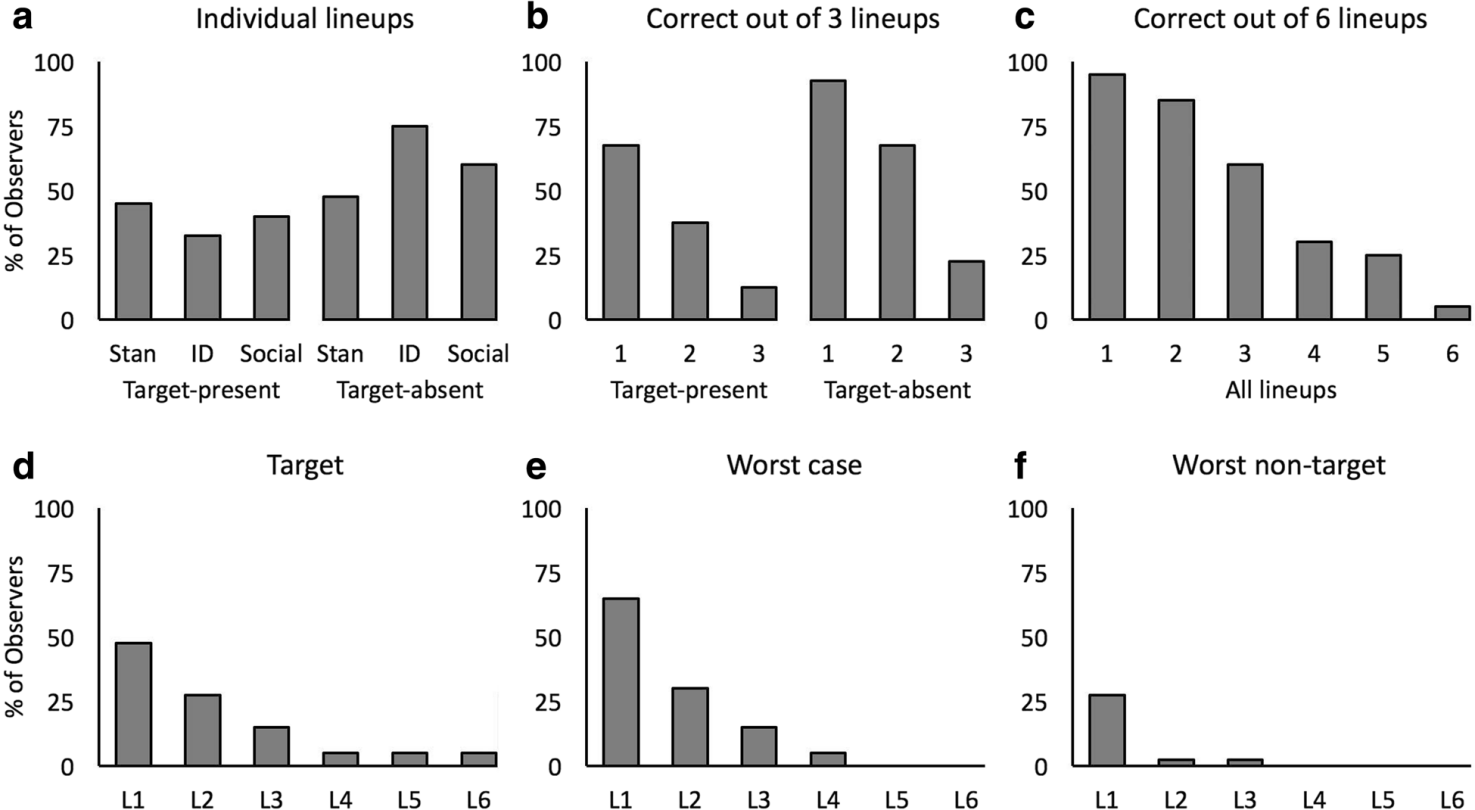
Illustration of observers’ responses in Experiment 2, showing hits for individual target-present and correct rejections for individual target-absent lineups (**a**), the number of hits and correct rejections out of three lineups (**b**), and the combined number of hits and correct rejections out of six lineups (**c**). In addition, observers’ combined hits and correct rejections were analyzed by adhering to the order in which the six lineups were seen, for the target (**d**), the worst case analysis (**e**), and for the worst non-target (**f**)

The data were analyzed in the same way as in Experiment 1. First, these data show that accuracy was once again error prone for individual lineups, as shown in Fig. 4a. Hits, for example, occurred only in 33–45% of target-present trials, whereas correct rejections accounted for 48–75% of responses to individual lineups. As in Experiment 1, a series of Wilcoxon signed-rank tests (with alpha corrected to .05/3 = .017 for three comparisons) showed no reliable differences in hits between the three target-present lineups or in correct rejections for the three target-absent lineups. A summary of these comparisons is provided in Table 2.

**Table 2:** A summary of statistical comparisons (Wilcoxon signed-rank test) for individual lineup accuracy and for accuracy out of three lineups in Experiment 2

Target-present			Target-absent		
Lineup	Accuracy	Wilcoxon	Lineup	Accuracy	Wilcoxon
Individual lineups					
Stan vs Social	45% vs 40%	Z = 0.50, *p* = .617	Stan vs Social	75% vs 48%	Z = 2.29, *p* = .022
Social vs ID	40% vs 33%	Z = 0.69, *p* = .491	Social vs ID	48% vs 60%	Z = 1.21, *p* = .225
Stan vs ID	45% vs 33%	Z = 1.67, *p* = .096	Stan vs ID	75% vs 60%	Z = 1.50, *p* = .134
Correct out of 3 lineups					
1 vs 2	68% vs 38%	Z = 3.46, *p* = .001^a^	1 vs 2	93% vs 68%	Z = 3.16, *p* = .002^a^
2 vs 3	38% vs 13%	Z = 3.16, *p* = .002^a^	2 vs 3	68% vs 23%	Z = 4.23, *p* < .001^a^
1 vs 3	68% vs 13%	Z = 4.69, *p* < .001^a^	1 vs 3	93% vs 23%	Z = 5.29, *p* < .001^a^

^a^Significant at *p* < .017, with alpha at .05 corrected for 3 comparisons

### Consistent lineup decisions in any order

We then analyzed how many observers were repeatedly correct across the three target-present lineups and the three target-absent lineups. These data are illustrated in Fig. 4b. For hits, a series of Wilcoxon signed-rank tests (with alpha corrected to .05/3 = .017 for three comparisons) showed that more observers identified the target from one than from two or all three target-present lineups. Correspondingly, more observers correctly rejected one target-absent lineup than two or all three (for a summary of these comparisons, see Table 2). This analysis was also conducted across all six lineups. This is illustrated in Fig. 4c and shows that 95% of observers acted correctly on the target at least once, but only 5% of observers were able to do so on all six lineups, *Z*(1, *N* = 40) = 6.00, *p* < .01.

### Consistent lineup decisions in actual order

The consistency of observers’ responses was also assessed as a function of the order in which the six lineups were shown, by calculating the percentage of observers who made a correct response on the *first* of the lineups and then carried on to do so without interruption on subsequent trials. These data are given in Fig. 4d and show that 48% of observers based their identification decisions on the target in Lineup 1, but only 5% of observers based their identification decisions consistently on the target across all six lineups, Z(1, *N* = 40) = 4.12, *p* < .01.

### “Worst case” analysis

As in Experiment 1, we also analyzed the repeated selection of non-target identities to contextualize repeated lineup decisions to the target. For this analysis, the first non-target that was selected by an observer was adopted as the “target” identity for that individual and all other lineup responses were then recoded accordingly. These data are provided in Fig. 4e and show that such non-target selections deteriorated from 65% in the first lineup to 0% when consistency was assessed across all six lineups, *Z*(1, *N* = 40) = 5.10, *p* < .01. In addition, a direct comparison of non-target and target selections showed that the percentage of these did not differ reliably for the first lineup seen (65% vs 48%), *Z*(1, *N* = 40) = 1.26, *p* = .21, or when consistency of these responses was assessed across all six lineups (0% vs 5%), *Z*(1, *N* = 40) = 1.41, *p* = .16.

### “Worst non-target” analysis

Once again, we also conducted a “worst non-target” analysis, by adopting the most frequently chosen non-target identity as the target. These data are provided in Fig. 4f and show that non-target selections declined from 28% in the first lineup to 0% across all six lineups, *Z*(1, *N* = 40) = 3.32, *p* < .01. While these worst non-target selections were less numerous than target selections after one lineup (28% vs 48%) and across all six lineups (0% vs 5%) on a descriptive level, these differences were not significant, *Z*(1, *N* = 40) = 1.51, *p* = .13 and *Z*(1, *N* = 40) = 1.41, *p* = .16, respectively.

## Discussion

This experiment examined the extent to which consistent target identifications are possible in a multiple-lineup procedure when faces vary more substantially in appearance than in Experiment 1. For this purpose, this experiment included standardized face photographs, as well as images from photo-identity cards and a social networking site. In comparison with Experiment 1, fewer hits were recorded (c.f. Figs. 2a and 4a), which indicates that the variability introduced by the new face images increased general task difficulty (e.g. Burton et al., 2005; Jenkins & Burton, 2011; Jenkins, White, Van Montfort, & Burton, 2011). Crucially, this manipulation also affected observers’ ability to act repeatedly on the target, with only 5% of individual observers capable of making correct eyewitness decisions across all six lineups. Moreover, the proportion of observers who made consistent identifications of the target and non-target identities did not differ. These findings converge with Experiment 1 to demonstrate that repeated identifications of the *same* unfamiliar person are very difficult, suggesting severe limitations in the facial representations that eyewitnesses can hold of such a perpetrator.

## Experiment 3

The preceding experiments demonstrate that repeated eyewitness identifications of the same target are difficult but also show that some observers can act more consistently on the target identity than others. This raises the question of what determines successful eyewitness repeat-identification of a target person. One possibility is that this reflects individual differences in the ability to recognize unfamiliar faces. Broad differences in this ability have been reported consistently between people, across a range of paradigms and image sets (e.g. Burton et al., 2010; Fysh & Bindemann, 2018; Megreya & Burton, 2006; Megreya & Bindemann, 2013; Russell, Duchaine, & Nakayama, 2009). Evidence is also accumulating that these individual differences are linked to eyewitness accuracy for a single lineup (see Bindemann et al., 2012; Geiselman et al., 2001; Hosch, 1994; Morgan et al., 2007). If these individual differences also underlie the variation among observers that is observed in Experiments 1 and 2, then performance on laboratory tasks testing the recognition of unfamiliar faces should relate to eyewitness identifications across multiple lineups for the same target.

To investigate this explanation, we created a laboratory version of the multiple-lineup paradigm reported in Experiments 1 and 2. In this task, observers viewed a video of a target person, which was then followed by three target-present and three-target-absent lineups for this person. This eyewitness task was then followed by two further laboratory tests for unfamiliar face recognition, comprising an adaptation of Bruce et al.’s (1999) 1-in-10 task and the Cambridge Face Memory Test (CFMT; see Duchaine & Nakayama, 2006). In the adaptation of the 1-in-10 task, observers were asked to memorize and then recognize a series of unfamiliar faces. On each trial of this test, observers studied a target face in isolation before determining its presence in a subsequent ten-person lineup. This 1-in-10 face-memory test produces large individual differences in identification performance (e.g. Megreya & Burton, 2006, 2008), which relate to eyewitness identification for a single lineup (Bindemann et al., 2012) and other internal factors, such as facets of observers’ personality (Megreya & Bindemann, 2013).

The CFMT also measures recognition memory for newly learned faces, which observers are then required to identify from a three-face array containing a target and two distractor faces. This test has been used widely to assess observers across the spectrum of face processing ability, such as those with impairments in face processing (e.g. Bobak, Parris, Gregory, Bennetts, & Bate, 2016; Ulrich, Wilkinson, Ferguson, Smith, Bindemann, et al., 2017; White, Rivolta, Burton, Al-Janabi, & Palermo, 2017) and those with exceptionally high ability (Bobak, Hancock, & Bate, 2016; Bobak et al., 2016; Russell et al., 2009). The 1-in-10 task and the CFMT therefore provide suitable tests against which performance on the multiple-lineup procedure can be compared.

As an additional aim for Experiment 3, we also sought to confirm that the pattern of results reported in the preceding experiments, which were based on only a single target identity, generalizes to other identities. We therefore examined the identification of two new targets in the multiple-lineup procedure. Both identities were also present in the lineups employed in Experiment 2, allowing us to retain these materials.

## Method

### Participants

Seventy-one undergraduate students (60 women, 11 men) from the University of Kent, with a mean age of 20 years (range = 18–34 years, *SD* = 3.3), participated in the experiment in return for course credit. All reported normal or corrected-to-normal vision.

### Materials and procedure

#### Multiple-lineup task

The same six lineups as in Experiment 2 were employed for the multiple-lineup procedure, with the exception that the target from the preceding experiments was replaced by another filler face who fit the same verbal description. This was due to the laboratory setting of this study and the original target being potentially familiar to participants at the University of Kent. In addition, two different identities from these lineups now served as targets (see Oriet & Fitzgerald, 2018). For these targets two short videos were recorded, which showed each person facing directly into the camera at a viewing distance of approximately 1 m before turning the head to the left and to the right. Each video lasted 30 s, with head movement duration standardized. In the experiment, every participant only viewed one of these videos. This was followed by a filler task requiring visual search for letters and numbers, which took approximately 5 min to complete. As in the preceding experiments, participants were then presented with six successive lineups (three target-present and three target-absent), which were shown in a random order. Participants were told there was an equal chance that the target would be present or absent in a lineup. They were asked to attempt to identify the target when he was present or to declare his absence when he was not. Once a lineup had been completed, it was removed from screen before the next lineup was presented. No time limit was given for the identification task.

#### 1-in-10 task

In the next phase of the experiment, participants were presented with 40 trials of the 1-in-10 task. In each trial, observers were first shown a video still of a single target face, depicting a full-face view with a neutral expression at a size of 4.1 cm (W) × 5.3 cm (H). Once observers were confident that they could identify the target, this was replaced by an identity lineup, consisting of ten full-face digital photographs at a size of 3.5 cm (W) × 4.5 cm (H). Thus, different images were used for initial exposure to a target and its counterpart in an identity lineup. Observers then had to indicate if the target was present in the lineup, and if so, identify who it is. Each observer was given 20 target-present and 20 target-absent trials in randomized order. A different target identity was used in each of these trials (for further details, see Bindemann et al., 2012).

#### Cambridge Face Memory Test

After the 1-in-10 task, participants completed the CFMT. The materials of the CFMT consisted of images of six male targets and 46 foil identities. Recognition memory for the targets was examined across several blocks. In the first block, participants studied three different orientations of a single target face for 3 s, before attempting to identify the target from a three-face array containing one of the study images and two distractor faces. This was repeated for each target. In the second block, observers studied six different but concurrent target faces for 20 s. They were then asked to identify a given target from a three-face array containing two distractor faces and a previously unseen view of a target. In a third block, which follows the procedure of Block 2, Gaussian noise was added to the face images to increase task difficulty (for further details, see Duchaine & Nakayama, 2006).

## Results

### Accuracy for individual lineups

**Fig. 5:**
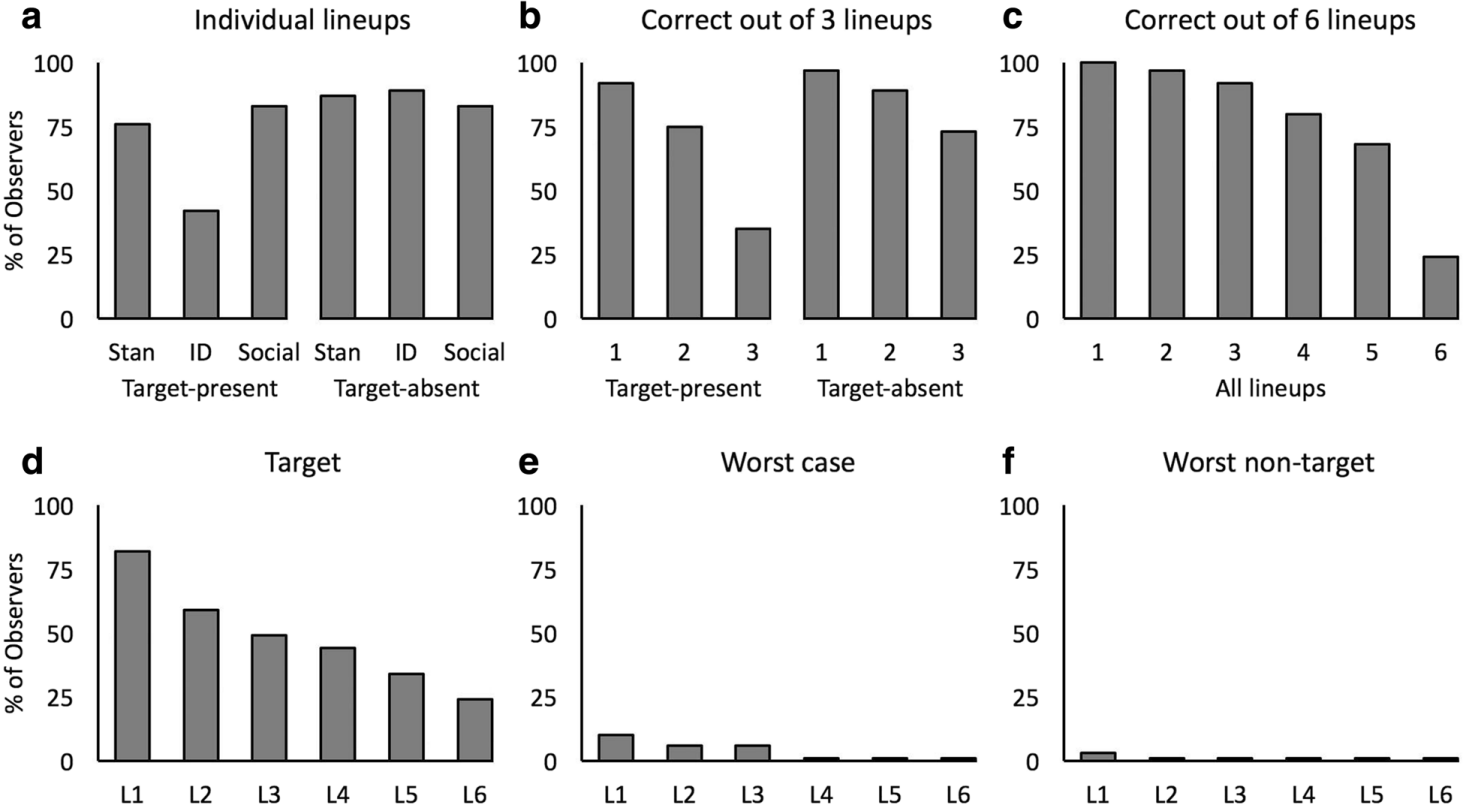
Illustration of observers’ responses in Experiment 3, showing hits for individual target-present and correct rejections for individual target-absent lineups (**a**), the number of hits and correct rejections out of three lineups (**b**), and the combined number of hits and correct rejections out of six lineups (**c**). In addition, observers’ combined hits and correct rejections were analyzed by adhering to the order in which the six lineups were seen, for the target (**d**), the worst case analysis (**e**), and for the worst non-target (**f**)

The data for the multiple-lineup procedure were analyzed in the same way as in the previous experiments (see Fig. 5). For brevity, we provide these data collapsed across both target identities, but data for individual targets is available as Supplementary Material. Across all lineups, hits occurred in 42–83% of target-present trials, whereas correct rejections accounted for 83–89% of responses, as shown in Fig. 5a. Wilcoxon signed-rank tests (with alpha corrected to .05/3 = .017 for three comparisons) showed that fewer hits were recorded with lineups comprising ID-card photographs than those constructed from face photographs from a social networking site, *Z*(1, *N* = 71) = 4.90, *p* < .01, and standardized photographs, *Z*(1, *N* = 71) = 4.38, *p* < .01. No other reliable differences in hits between the three target-present lineups or correct rejections for the three target-absent lineups occurred. A summary of these comparisons is provided in Table 3.

**Table 3:** A summary of statistical comparisons (Wilcoxon signed-rank test) for individual lineup accuracy and for accuracy out of three lineups in Experiment 3

Target-present			Target-absent		
Lineup	Accuracy	Wilcoxon	Lineup	Accuracy	Wilcoxon
Individual lineups					
Stan vs Social	76% vs 83%	Z = 1.29, *p* = .197	Stan vs Social	87% vs. 83%	Z = 0.83, *p* = .405
Social vs ID	83% vs 42%	Z = 4.90, *p* < .001^a^	Social vs ID	83% vs 89%	Z = 1.41, *p* = .157
Stan vs ID	76% vs 42%	Z = 4.38, *p* < .001^a^	Stan vs ID	87% vs 89%	Z = 0.28, *p* = .782
Correct out of 3 lineups					
1 vs 2	92% vs 75%	Z = 3.46, *p* = .001^a^	1 vs 2	97% vs 89%	Z = 2.45, *p* = .014^a^
2 vs 3	75% vs 35%	Z = 5.29, *p* < .001^a^	2 vs 3	89% vs 73%	Z = 3.32, *p* = .001^a^
1 vs 3	92% vs 35%	Z = 6.33, *p* < .001^a^	1 vs 3	97% vs 73%	Z = 4.12, *p* < .001^a^

^a^Significant at *p* < .017, with alpha at .05 corrected for 3 comparisons

### Consistent lineup decisions in any order

We then analyzed how many observers were repeatedly correct across the three target-present and three target-absent lineups. These data are illustrated in Fig. 5b. As previously, Wilcoxon signed-rank tests showed that more observers identified the target from one than from two or all three target-present lineups. Similarly, correct rejections were highest for one than two or all three target-absent lineups (for a summary for these comparisons, see Table 3). This analysis was also conducted across all six lineups. This is illustrated in Fig. 5c and showed that although all (i.e. 100%) of observers acted correctly on the target at least once, only 24% of observers did so on all six trials, *Z*(1, *N* = 71) = 7.35, *p* < .01.

### Consistent lineup decisions in actual order

The consistency of observers’ responses was assessed again as a function of the order in which the six lineups were shown. These data are given in Fig. 5d and show that the percentage of observers who made a correct eyewitness decision fell from 82% in Lineup 1 to 24% by Lineup 6, *Z*(1, *N* = 71) = 6.40, *p* < .01.

### “Worst case” analysis

As in preceding experiments, the repeated selection of non-target lineup identities was also examined. These data are provided in Fig. 5e and show that non-target selections were at 10% in the first lineup but were made consistently across all six lineups by only 1% of observers, *Z*(1, *N* = 71) = 2.45, *p* < .05. A direct comparison of target and non-target selections showed that the percentages of these differed substantially for Lineup 1 (82% vs 10%), *Z*(1, *N* = 71) = 6.76, *p* < .01, and across all six lineups (24% vs 1%), *Z*(1, *N* = 71) = 3.77, *p* < .01.

### “Worst non-target” analysis

We again conducted a “worst non-target” analysis. For each of the two targets, we adapted the most frequently misidentified non-target as the target and then recalculated identification performance on this basis. These data are provided in Fig. 5f and show that non-target selections were at 3% in Lineup 1 and fell to 1% by Lineup 6, but this small difference was not significant, *Z*(1, *N* = 71) = 1.00, *p* = .32. The proportion of target and worst non-target selections differed in Lineup 1 (82% vs 3%), *Z*(1, *N* = 71) = 7.35, *p* < .01, and across all six lineups (24% vs 1%), *Z*(1, *N* = 71) = 3.77, *p* < .01.

### Correlations with the 1-in-10 task and CFMT

To explore whether differences in accuracy between participants on the multiple-lineup task are linked to individual differences in the ability to recognize unfamiliar faces, Pearson’s correlations between performance on the lineup task and the 1-in-10 task and CFMT were examined. The CFMT utilizes a forced-choice methodology. Therefore, only hit data are attainable. Hits on the multiple-lineup task, averaged across the three target-present lineups, correlated with the percentage of hits on the 1-in-10 task, *r*(69) = .43, *p* < .05, and the total score on the CFMT, *r*(69) = .31, *p* < .01, with effect sizes being small to moderate. Correct rejections for the multiple-lineup task and the 1-in-10 task showed no significant correlation, *r*(69) = .19, *p* = .12.

## Discussion

This experiment replicates the main findings of the preceding experiments by showing that observers find it difficult to consistently act on a target identity across six lineups of faces. For example, whereas all observers (100%) were accurate in at least one of the lineups, by making a correct identification or lineup rejection, only 24% of observers were able to do so across all six lineups. However, consistent identifications of non-target identities were even less likely across all six lineups, being made by only 1% of all observers.

Experiment 3 extends the findings of Experiments 1 and 2 in two ways. First, this experiment obtained these effects with two new target identities and under more controlled laboratory conditions than the field study paradigm of Experiments 1 and 2, demonstrating generalizability of these effects. Second, this experiment showed correlations of individual performance on the multiple-lineup task with two established tests of unfamiliar face recognition, comprising the 1-in-10 task (Bruce et al., 1999) and CFMT (Duchaine & Nakayama, 2006). These correlations were small to moderate in strength, indicating that one likely source of variation in performance across observers in the multiple-lineup procedure reflects individual differences in the ability to process unfamiliar faces.

These findings converge with other studies reporting correlations between eyewitness accuracy and tests of face recognition ability (e.g. Geiselman et al., 2001; Hosch, 1994; Morgan et al., 2007), including some that have utilized the same 1-in-10 task (Bindemann et al., 2012). As in Bindemann et al. (2012), this correlation was present for lineup identifications but not rejections. One possible explanation for the absence of this latter correlation could be that “target absent” responses may reflect two different response categories in this paradigm, comprising observers’ explicit knowledge that a target is absent from a lineup (i.e. a correct rejection) or rejection of a lineup when observers simply do not know whether a target is present or not (i.e. a “don’t know” response; Sauerland, Sagana, & Sporer, 2012).

## General discussion

This study investigated the ability of individual eyewitnesses to act consistently on a target identity across multiple lineups, comprising three target-present and three target-absent lineups. Performance was error-prone for individual lineups. For example, only 45% of observers made a correct decision to the first lineup in Experiment 1. A more striking pattern emerged when individuals’ ability to consistently identify the presence of a target and to determine his absence from a lineup was explored. When highly similar target images were shown across lineups in Experiment 1, following a face-to-face interaction in a field paradigm, only 28% of observers based their decision correctly on the target identity across all lineups. This substantially exceeded consistent identification errors, which were made by acting on a non-target identity across the six lineups and were committed by 5% of observers. However, the ability to act consistently on a target identity reached similarly low levels when greater within-person variability was introduced in the lineup faces in Experiment 2. Under these conditions, 48% of observers made a correct decision to the first lineup, but only 5% of participants were correct across all six lineups, and none of the observers (0%) acted with such consistency on a non-target.

Experiment 3 extended these findings to a laboratory setting and different target identities. This experiment utilized the same high-variability multiple lineups as Experiment 2, but exposed participants to controlled close-up video of the target faces. In this paradigm, accuracy for individual lineups was improved compared to Experiment 2. For example, 82% of observers now made a correct decision to the first lineup and consistent target decisions, across all six lineups, were made by 24% of participants. These results indicate that consistent correct target decisions need not be as infrequent as Experiment 2 suggests and could reflect the more controlled conditions of the laboratory eyewitness test or enhanced accuracy for the new targets that were employed in this study. Crucially, however, Experiment 2 replicated another key finding of the preceding experiments, by demonstrating that consistent decisions to non-target identities were even less frequent, at 1% across all six lineups.

These data represent an important finding. The ability to recognize a face repeatedly, and across variable images, is a hallmark of the accurate identification of familiar faces (see, e.g. Bruce & Young, 1986; Burton et al., 2005), but the extent to which this is possible with unfamiliar faces is less clear. Recent studies have shown that repeat identifications of unfamiliar faces are taxing in identity-matching tasks (e.g. Alenezi & Bindemann, 2013; Bindemann et al., 2012; Bindemann & Sandford, 2011). The current study demonstrates that this is also the case in an eyewitness scenario that requires the repeated *recognition* of a face from memory. Indeed, this task appears impossible for almost all individual observers when face images display more realistic within-person variability in conjunction with the naturalistic eyewitness encounter of the field study paradigm of Experiment 2. This indicates that the stored facial representations that observers could develop in the eyewitness scenario here do not necessarily allow for the robust identification of targets. In turn, Experiment 3 shows that identifications can occur more frequently with the same lineup images under more controlled conditions.

A question that arises is what determined successful repeat-identifications in these experiments in some observers but not others. One possibility is that these differences are linked to observers’ inherent ability to process unfamiliar faces (Wilmer et al., 2010; Zhu et al., 2010). Some individuals may be naturally capable of being better eyewitnesses, perhaps akin to “super-recognizers,” who have superior levels of face recognition ability (e.g. Noyes, Phillips, & O’Toole, 2017). In support of this reasoning, eyewitness accuracy in the multiple-lineup task of Experiment 3 correlated with the 1-in-10 task of unfamiliar face recognition as well as the CFMT, which has been employed consistently as a measure of super recognition (e.g. Bobak, Bennetts, Parris, Jansari, & Bate, 2016; Bobak et al., 2016; Russell et al., 2009). However, these correlations were only small to moderate in strength and therefore cannot explain the pattern of results alone.

Another possibility is that the individual variation in the multiple-lineup procedure also reflects qualitative differences in the initial interaction with the target, such as the attention that individual observers paid to the target’s face. It has been shown, for example, that fixations on a perpetrator’s face relate to eyewitness accuracy under laboratory conditions that provide participants with the same crime (video) content (Attard & Bindemann, 2014). Such findings could perhaps also explain why accuracy improved in Experiment 3, which provided all participants with exactly the same video material for initial exposure to the targets, compared with the inevitably more variable exposure that was provided by the social interaction of the field study paradigm in Experiment 2 (cf. Figs. 4 and 5). Future studies are required to further clarify the factors that determine individual differences in multiple-lineup performance.

### Implications

Eyewitness identification, in research and practice, is typically assessed via a single person identification. Without prior knowledge of the true target identity in the lineup, as is the case in criminal investigations, it is difficult to confidently assess the accuracy of such eyewitness responses (e.g. Wells, Memon, & Penrod, 2006; Wells & Olson, 2003). The current experiments confirm that a single eyewitness identification can be misleading, for two reasons. First, many individuals failed to either identify a target from a lineup or to correctly identify its absence. Second, most individuals made a correct decision to any given lineup but failed to do so consistently across all six lineups. In light of these findings, we would argue that a test of a person’s memory of a culprit must provide insight into the *degree* of familiarity that an eyewitness has gained with a target to establish their accuracy. An individual’s ability to act on a target consistently appears to provide a potential index for this purpose.

A multiple-lineup procedure with faces has the potential to be developed into such a solution for applied settings and could provide some parsimony between face recognition theory and eyewitness identification in practice. As noted earlier, cognitive theories have stipulated for considerable time that the recognition of a familiar person should be triggered by any image of a face (e.g. Bruce & Young, 1986; Burton et al., 2005; Schweinberger & Burton, 2003). According to these theories, repeated identification of someone from different images should therefore present an index of the degree to which familiarity with this person exists. Considering that eyewitness identification is essentially a test of the familiarity that a person has acquired with the appearance of a perpetrator, we would suggest that such standards should be considered to understand recognition accuracy also in this domain.

We draw these conclusions with caution considering that this is only the first study to directly examine eyewitness identification with multiple lineups of faces. Furthermore, whereas correct decisions were much more likely for targets than non-targets across six lineups in Experiments 1 and 3, we note that the proportion of observers that exhibited such consistent behavior was very low, and comparable, for the target and non-targets in Experiment 2 (at 5% and 0%, respectively). From an applied perspective, further work is clearly needed to resolve why consistent target decisions are generally low under some conditions and how the proportion of these decisions could be increased for practical use.

In practice, a multiple-lineup approach could also create another conundrum with regard to the *number* of eyewitnesses that remain useful in criminal investigations after the completion of a multiple-lineup procedure. In Experiment 1, for example, we found that 45% of observers made a correct decision to the first lineup, but only 28% were also consistently accurate across all six lineups. The repeated assessment of eyewitness accuracy could therefore lead to the exclusion of a great number of individuals that would appear to be good eyewitnesses by current standards. While this data loss could be reduced by decreasing the number of repeat-identifications, it raises the question of how we should define a “good eyewitness” more generally. The current findings could suggest that such a definition must be applied with flexibility. A single lineup will provide a greater pool of “good” eyewitnesses, who *may* be accurate, which would be advantageous during the search for perpetrators during criminal investigations. However, based on the current data we would argue that such an inclusive approach can only provide limited information about the *actual* familiarity of a witness with a target individual. The inclusive approach is therefore of debatable use for establishing the identity of a target confidently during judicial proceedings (e.g. Lindsay et al., 1987; Pryke et al., 2004; Sauerland & Sporer, 2008; Sauerland et al., 2013). Under such conditions, the reduction of “good” eyewitnesses in a multiple-lineup procedure might be considered a data gain, by offering greater precision in establishing the memory of individual eyewitnesses for a perpetrator.

## Conclusions

In summary, the current experiments provide evidence that repeated eyewitness identifications of the same target from multiple lineups are taxing in laboratory and field paradigms. This problem is such that when different images of the target person display realistic within-person variability and are used in conjunction with a field paradigm, repeat identifications appear impossible for almost all eyewitnesses. These findings suggest that most eyewitnesses only develop limited cognitive representations of a target that do not allow for robust identification. In turn, the ability of individual eyewitnesses to repeatedly act on a target should provide insight into the degree of familiarity they have acquired with this person, and could therefore provide a potential index of eyewitness accuracy.

## Additional file

Additional file 1: Supplementary Material: Full Datasets for Experiments 1, 2 and 3. (XLSX 43 kb)

## Abbreviations

CFMT: Cambridge Face Memory Test
